# Use of point-of-care ultrasound (POCUS) in French urgent primary care: a national survey of general practitioners in the SOS Médecins network

**DOI:** 10.1186/s12875-026-03393-4

**Published:** 2026-05-27

**Authors:** Nawele Boublay, Pascal Rippert, Touria Hajri, Julien Berthiller, Anne-Marie Schott-Pethelaz, Pauline Bertois

**Affiliations:** 1 Université Claude Bernard Lyon 1, Lyon, France; 2 Service Recherche et Epidémiologie Cliniques, Pôle Santé Publique, Hospices Civils de Lyon, Lyon, France; 3 Research on Healthcare Performance RESHAPE, INSERM U1290, Université Claude Bernard Lyon 1, Lyon, France; 4 Commission scientifique SOS Médecins France FR, Paris, France

**Keywords:** Point-of-care ultrasound, POCUS, Prevalence, General practice, Primary care, Urgent care, France, SOS Médecins

## Abstract

**Background:**

Point-of-care ultrasound (POCUS) is increasingly recognized as a valuable diagnostic tool in general practice. However, its actual use among French general practitioners (GPs) working in urgent care settings, such as SOS Médecins, a nationwide network of GPs providing home-based urgent primary care 24/7, remains poorly documented.

**Objectives:**

To assess the prevalence, modalities of use, self-reported perceived impact, and barriers to the adoption of POCUS among GPs working in the SOS Médecins network across France.

**Methods:**

A national cross-sectional online survey was distributed to all eligible SOS Médecins GPs. The questionnaire addressed POCUS usage, clinical indications, equipment access, perceived benefits, training needs, and adoption barriers. Descriptive statistics were used, and comparisons between users and non-users were performed using chi-square or Fisher’s exact tests.

**Results:**

Among 211 respondents, 47.9% reported current POCUS use. Statistically significant differences between users and non-users were observed for age group (*p* = 0.021) and years of experience in SOS Médecins (*p* = 0.003). The most frequent indications included abdominal pain (79.2%), vascular assessment (76.2%), and respiratory symptoms (65.3%). Most users relied on portable devices (69.8%) and performed focused, indication‑driven scans (87.4%). POCUS was perceived as having a positive or very positive impact on triage (86.0%), treatment decisions (81.7%), faster diagnosis (67.0%), professional autonomy (59.0%), and the doctor–patient relationship (50.0%). Nevertheless, only 15.6% of users reported billing for POCUS acts. Among non-users, the main barriers were lack of specific training (63.6%), limited time (44.5%), equipment cost (40%), and doubts about clinical utility (23.6%). More than half (54.5%) expressed interest in receiving training.

**Conclusion:**

POCUS is currently used by nearly half of SOS Médecins GPs providing urgent primary care in France. Despite strong clinical interest and perceived utility, adoption remains limited by training gaps, structural barriers, and lack of clear financial incentives.

Future national strategies should prioritize the development and structuring of undergraduate POCUS education in medical schools, alongside expanded continuing training opportunities, improved financial recognition, and local mentorship initiatives, to support equitable and sustainable integration into French primary care.

## Introduction

Point-of-care ultrasound (POCUS) is increasingly recognized as a valuable diagnostic modality across both emergency and primary care settings. POCUS, defined as a clinician-performed bedside imaging tool to address focused clinical questions, enhances physical examination, expedites diagnostic decisions, and reduces dependency on traditional imaging modalities [1–3]. When systematically integrated into general practice workflows, POCUS has also been associated with greater diagnostic precision, improved triage decisions, and safer, more efficient patient management [4, 5].

In primary care, studies from European countries such as Norway or Switzerland have documented the growing integration of POCUS into routine general practice supported by dedicated strategies, evolving reimbursement models, and improved access to portable devices [6–9].

In Norway, a national reimbursement‑based register study reported that approximately 30% general practitioners (GPs) had claimed at least one POCUS procedure in 2016 [7]. In Switzerland, a cross-sectional survey among primary care physicians found that the most commonly self-performed ultrasound scans were abdominal (57.9%) and musculoskeletal (22.0%) examinations [6]. Together, these national experiences reflect a broader trend: the progressive shift of ultrasound from hospital-based radiology units to outpatient and community care, where GPs are developing new practice models and overcoming structural barriers [10].

In France, however, the diffusion of POCUS into general practice remains limited. While several medical theses have explored GPs’ perceptions and training needs regarding POCUS, no peer-reviewed national data are currently available on its actual use in urgent primary care of its role in ensuring continuity of care.

However, significant barriers persist in the French medical context. These include the absence of ultrasound training during undergraduate and postgraduate medical education, lack of formal recognition or information about remuneration for POCUS acts, equipment costs, and time constraints during consultations [11, 12]. As a result, the implementation of POCUS within French general practice remains slow and inconsistent.

SOS Médecins is a nationwide network of approximatively 1,300 GPs providing 24/7 urgent and unplanned primary care, primarily through home visits, across more than 60 locations in France. This unique organization plays a key role in the out-of-hospital management of acute conditions, particularly among older or frail populations [13]. Although SOS Médecins GPs are fully qualified GPs, with the same initial medical training, certification and professional values as office-based GPs, their clinical practice differs in several key aspects. SOS Médecins activity is primarily centered on home visits rather than office consultations, focuses on unscheduled and urgent care, and is oriented toward acute clinical events rather than longitudinal follow-up. Importantly, SOS Médecins GPs care for the same patient population as community GPs and operate within the same primary care system, ensuring first-contact care and adapting to population needs, particularly when the patient’s usual GP is unavailable. Their practice is therefore complementary to that of office-based GPs and contributes to the continuity of care within the French primary care system. As such, SOS Médecins offers a critical setting to evaluate POCUS integration. These GPs often work in unscheduled, home-based contexts where patients present with acute symptoms, such as abdominal pain, dyspnea, or trauma, and lack immediate access to radiological assessment. In such situations, POCUS could provide timely diagnostic information to guide early clinical decisions [11, 14].

While some local projects have emerged, mainly in the form of unpublished medical theses or informal pilot programs, no unified national strategy presently exists to promote structured POCUS training and sustainable integration into French general practice. This challenge is not exclusive to France, as similar situation has been reported in other European settings, including Scandinavia, where training programs have demonstrated clinical value but have not yet resulted in widespread daily use among GPs [15].

This study aims to assess the prevalence, modalities of use, and self-reported perceived impact of POCUS among GPs providing urgent medical serviceat a national level. It also explores how the tool is used (devices, indications, frequency), practitioners’ perceptions, and barriers to broader adoption in this unique care setting.

## Methods

### Study design and objectives

We conducted a nationwide, cross-sectional, descriptive study between March 2025 and September 2025. The primary objective was to describe the prevalence of point-of-care ultrasound (POCUS) use among GPs working within the SOS Médecins network in France. Secondary objectives included identifying the modalities of POCUS use, perceived barriers and facilitators, and the characteristics of practitioners using POCUS in routine practice.

### Setting and population

SOS Médecins is a nationwide network of approximately 1,300 GPs providing 24/7 urgent and continuity of primary care in France, primarily through home visits. The study targeted all GPs actively practicing within the SOS Médecins France network at the time of the survey.

### Sample size

The eligible population consisted of approximately 1,300 GPs. No a priori sample size calculation was performed, as no prior national data were available to inform a specific hypothesis of interest or expected response rate. Incomplete or clearly inconsistent responses were excluded from analysis. A total of 216 GPs initially responded to the survey. Five questionnaires were excluded due to incomplete data or internal inconsistencies regarding POCUS use status, resulting in a final analytical sample of 211 respondents.

### Data collection

An anonymous, structured, self-administered questionnaire was developed based on existing literature and expert input. The survey comprised 37 items covering the following domains:

- Demographics and professional characteristics
- Current access to and use of POCUS
- Clinical indications and anatomical targets
- Self-reported perceived impact on clinical decision-making
- Training history and learning needs
- Barriers and facilitators to POCUS adoption

Practice environment (urban, peri-urban, rural) and access to radiological services were self-reported by respondents and not based on predefined administrative or geographic criteria. Selection of clinical indications and anatomical targets was guided by international studies, ensuring comparability with prior research and enhancing generalizability [8, 9, 16, 17].

The self-reported perceived impact of POCUS on patient management (including time-saving, diagnostic assistance, and therapeutic decision-making) was assessed using a 5-point Likert scale, ranging from “1-Very negative opinion” to “5-Very positive opinion “, as recommended in previous studies [18, 19].

The questionnaire was built and distributed via a secure online platform (RedCap©) [20]. The questionnaire items were based on single-choice lists, except for the questions related to practice environment, clinical indications, anatomical targets, training, and barriers and facilitators—which were multiple-choice lists—with the addition of comment sections.

GPs were first contacted by email via the national SOS Médecins mailing list. Two reminder emails were sent at three-week intervals. Additionally, the study was presented at the SOS Médecins national congress in June 2025, where a QR code was displayed to facilitate voluntary participation. Participation was voluntary and without financial compensation.

### Statistical analysis

Data were analyzed using SAS software, version 9.4 (SAS Institute Inc., Cary, NC, USA). Descriptive statistics were used to summarize participant characteristics and response distributions. Categorical variables were described by frequencies and percentages. Chi-square or Fisher’s exact tests were used for comparisons where relevant, with a significance threshold of *p* < 0.05.

## Results

### Population characteristics

GPs characteristics are provided in Table 1. Out of approximately 1,300 GPs contacted, 211 completed the questionnaire (16%): 110 non-users of point-of-care ultrasound (POCUS) and 101 current users. The majority were male in both groups (80.9% among non-users, 82.2% among users). Age distribution differed significantly between users and non-users (*p* = 0.021). POCUS users were mainly in mid‑career (35–44 years; 43.6% vs. 30.9%), whereas non‑users were more often at the beginning (< 35 years; 20% vs. 14.9%) or end (≥ 65 years; 10.9% vs. 0.99%) of their career.

**Table 1:** Participant characteristics by use of point-of-care ultrasound (POCUS) among SOS Médecins general practitioners

Variable	Modality	POCUS users (*n* = 101) N (%)	Non-users (*n* = 110) N (%)	*p*-value
Age group (years)	25–34	15 (14.9%)	22 (20.0%)	
	35–44	44 (43.6%)	34 (30.9%)	
	45–54	21 (20.8%)	21 (19.1%)	
	55–64	20 (19.8%)	21 (19.1%)	
	≥ 65	1 (0.99%)	12 (10.9%)	**0.02**
Gender	Male	83 (83%)	89 (80.9%)	
	Female	17 (17%)	21 (19.1%)	0.69^#^
Years in SOS practice (years)	< 5	18 (17.8%)	35 (31.8%)	
	5–10	29 (28.7%)	17 (15.5%)	
	11–20	35 (34.7%)	25 (22.7%)	
	> 20	19 (18.8%)	33 (30.0%)	**0.003**
Practice environment*	Urban	90 (89.1%)	100 (90.9%)	
	Peri-urban	49 (48.5%)	39 (35.5%)	
	Rural	14 (13.9%)	14 (12.7%)	-
Access to radiologist	Easy	31 (30.7%)	43 (39.1%)	
	Moderate	60 (59.4%)	55 (50.0%)	
	Difficult	10 (9.90%)	12 (10.9%)	0.37

Bold values indicate statistically significant differences (*p* < 0.05)

*Practice environment data were collected using a multiple-choices list

^#^One participant selected ‘Other / No answer’ for gender; this response was excluded from group comparison due to insufficient sample size

A significant difference was also observed regarding years of experience in SOS Médecins (*p* = 0.003). Mid-career GPs (5–20 years) were more likely to use POCUS (63.4%), while early-career and senior doctors were more represented among non-users (31.8% and 30.0%, respectively).

Practice settings were not different across groups, with most respondents working in urban or peri-urban areas (urban: approximatively 90% for both users and non-users; peri-urban: 35.5% non-users vs. 48.5% users). Access to radiologists was not significantly different (*p* = 0.374), with most describing it as moderate or difficult (non-users: 69.9%; users: 69.3%).

### Use and non-use of POCUS

#### Current users (*n* = 101)

Most users had been practicing POCUS for more than 5 years (37.5%), followed by 1–3 years (32.3%) and 3–5 years (21.9%). Daily use was reported by 41.1%, while 33.7% used it weekly. The majority (87.4%) reported performing focused scans for specific clinical indications, rather than general imaging.

#### Non-users (*n* = 110)

Non-users accounted 52.1% of respondents. When asked whether specific training might encourage adoption, 54.5% responded affirmatively, 27.3% were unsure, and 18.2% indicated no interest.

Further details on reported barriers to adoption, including perceived usefulness, time constraints, and access or equipment issues, are presented in Sect.  6 (Barriers to Use and Training Gaps).

### Indications and anatomical targets

**Fig. 1:**
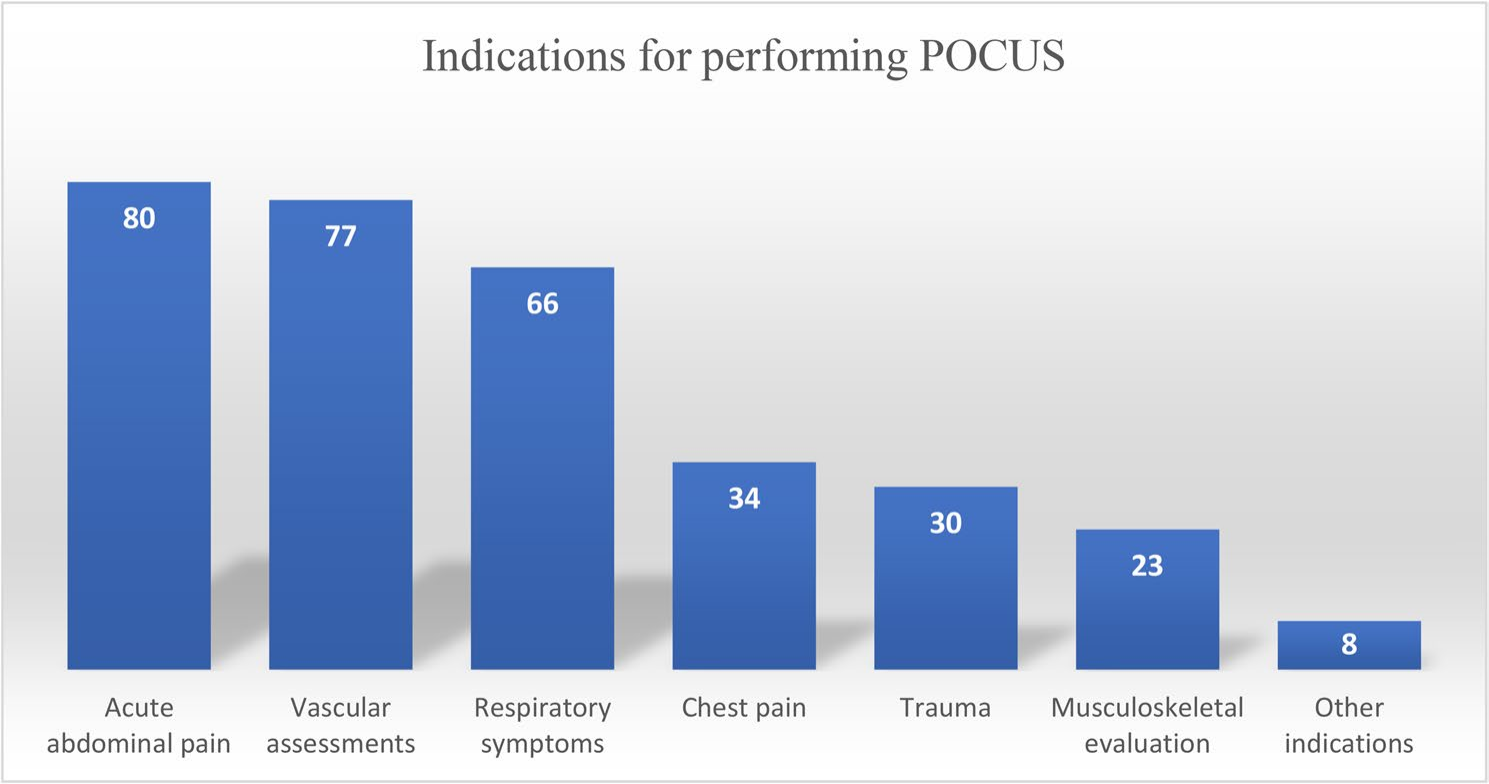
Clinical indications for point-of-care ultrasound among current users (*n* = 101). Values represent number of respondents

Among users, the most common indications for using POCUS were Abdominal pain (79.2%), Vascular assessments (76.2%), Respiratory symptoms (65.3%), Chest pain (33.7%), Trauma assessment (29.7%) and Musculoskeletal evaluation (22.8%) (Fig. 1).

Additional uses reported in open comments included bladder volume evaluation, gynecological indications (e.g. suspected ectopic pregnancy, ovarian cyst), as well as ocular, thyroid, and testicular assessments.

### Access to equipment

Among POCUS users, 69.8% of POCUS users relied on portable ultrasound devices, while 12.5% used fixed devices and 17.7% reported using both.

Among non-users, access to equipment was rarely perceived as a major barrier with only 2.7%, citing difficulty accessing an ultrasound device, whereas high equipment cost was reported by 39.6%.

### Self-reported perceived impact of POCUS

**Fig. 2:**
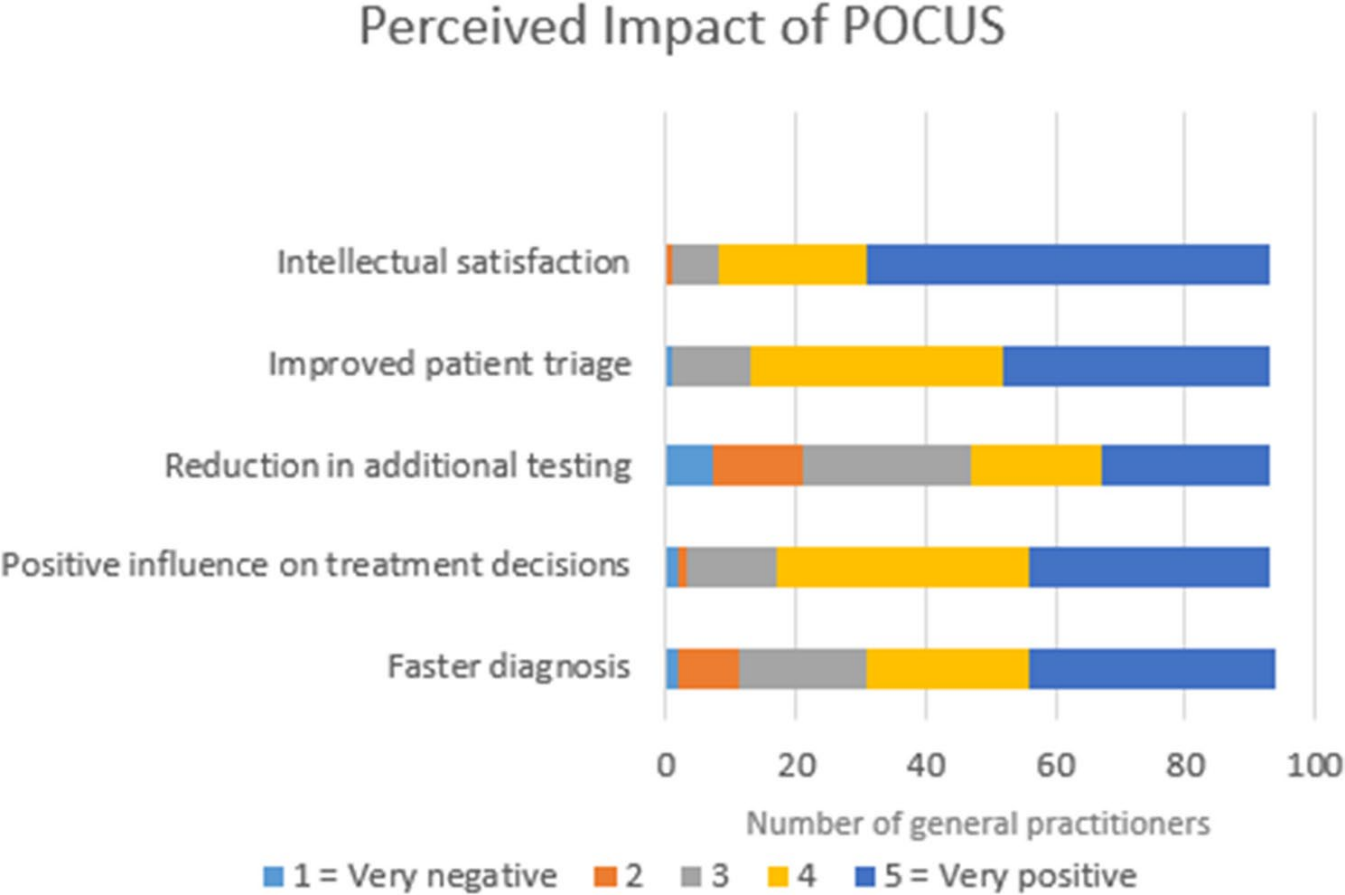
Self-reported perceived impact of POCUS were made among current users (*n* = 101) on a 5-point Likert scale (1 = very negative; 5 = very positive). Missing responses were excluded

The perceived clinical value of POCUS was high among users across all assessed dimensions. As shown in Fig. 2, the majority of respondents rated its impact as positive or very positive on intellectual satisfaction (91.4%), patient triage (86.0%), faster diagnosis (67.0%), and treatment decisions (81.7%). The self-reported perceived impact on reducing additional testing was slightly lower (49.5%).

Additionally, qualitative comments reinforced this favorable perception. For example, one participant described POCUS as “the stethoscope of the 21st century,” while another stated: “It has revolutionized my practice and decision-making processes.”

Notably, even among non-users, 57.3% of respondents rated the potential impact of POCUS as positive or very positive, indicating a strong underlying interest.

### Barriers to use and training gaps

**Fig. 3:**
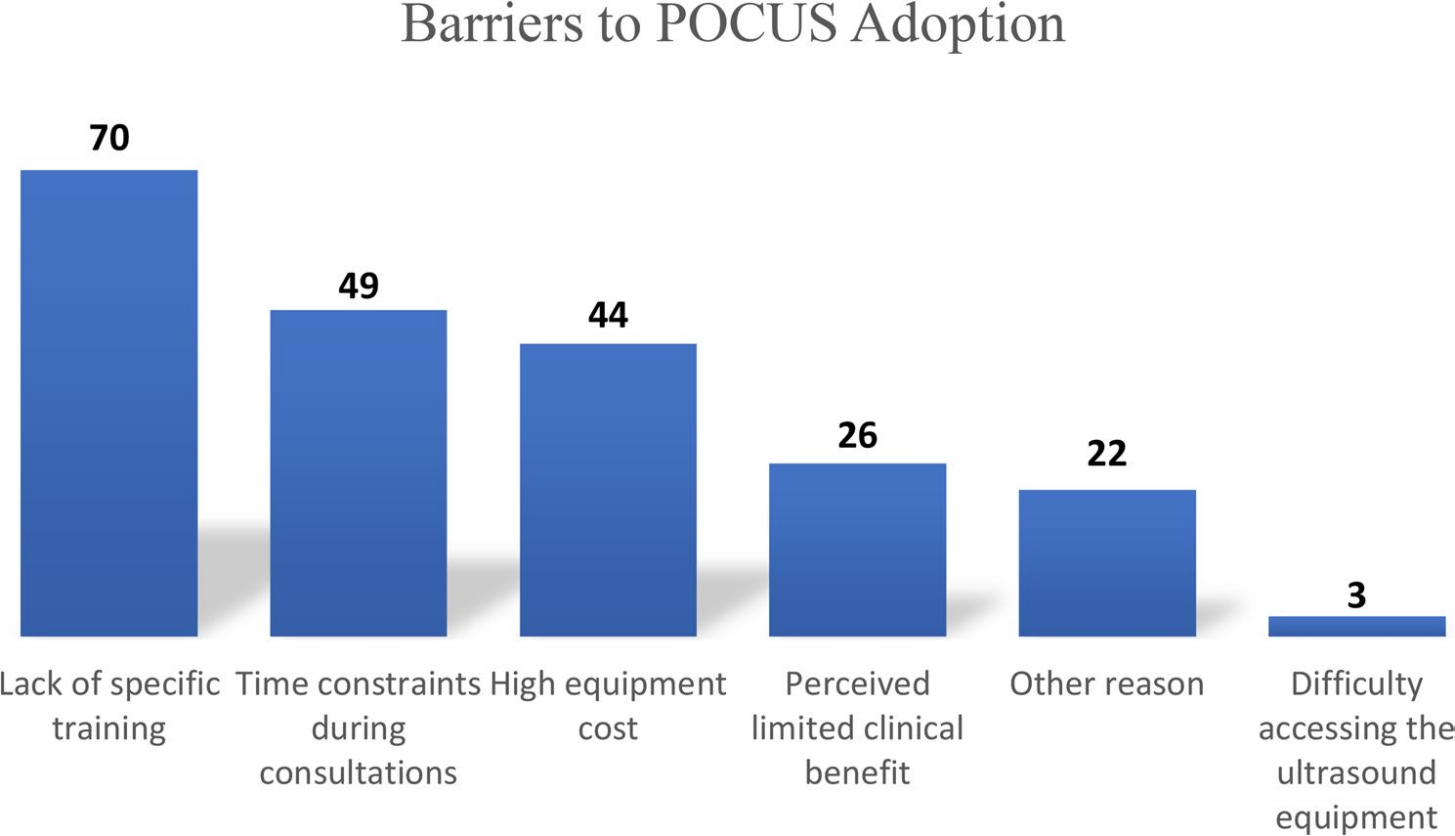
Reported barriers to adopting POCUS among non-users (*n* = 110). Values represent number of respondents.

Among non-users, the main barriers included Lack of training (63.6%), Lack of time (44.5%), Equipment cost (40%) and Perceived low utility (23.6%) (Fig. 3).

Free-text responses also highlighted recurring concerns about medico-legal uncertainties, technical proficiency, and the absence of billing incentives, all of which further discouraged adoption.

Among users, 89.5% reported having some form of training, including a University or Inter-university Diploma in POCUS (22.8%), industry-sponsored sessions (15.8%), continuing medical education (61.4%), and self-directed learning (29.7%).

Yet only 15.6% reported integrating POCUS into their billing practices.

### Suggestions and open comments

Approximately one-quarter of respondents (25.1%, *n* = 53) provided qualitative comments, offering rich insights into both clinical practice and system-level challenges.

Six major themes emerged:

- Clinical utility and evolving standards: Several respondents described POCUS as “indispensable” or “revolutionary” in acute care contexts. Comments emphasized its role in ruling out life-threatening conditions, enhancing diagnostic speed, and improving outpatient triage: “It is the stethoscope of the 21st century.” “I can’t work without it anymore.”
- Training needs and mentorship: Many called for structured, hands-on training pathways tailored to the SOS Médecins context. Proposals included internal mentorship programs, partnerships with existing certified trainers, and echo-first initiatives modelled on local examples: “The best training I had was from fellow SOS physicians.” “Why not have internal training by experienced DU-certified colleagues?”
- Institutional and financial recognition: A dominant theme was the demand for a dedicated billing code, analogous to those available for electrocardiography (ECG), and better guidance regarding the French medical classification system for procedures and billing (Classification Commune des Actes Médicaux- CCAM): “A specific billing code would recognize the time, cost and skill invested.” “We need clearer guidelines on how to code ultrasound procedures.”
- Equipment costs and technical barriers: Respondents frequently cited the high cost of quality devices and the difficulty of fitting scans into short home visits. “If only performant machines weren’t so expensive.” “Adds 15 + minutes per consultation, hard to manage in urgent care.”
- Professional identity and patient rapport: Several comments highlighted how POCUS reinforces clinical credibility, fosters patient trust, and preserves GPs’ central role in a shifting care landscape: “It boosts patient trust and strengthens the doctor-patient bond.” “We must stay central in the system. POCUS helps us remain indispensable.”
- Strategic vision for the future: Beyond individual practice, a number of respondents emphasized the need for a national strategy, equipment supports from the network, and a shared vision for integrating POCUS sustainably in out-of-hospital care. “POCUS is a necessity, not an option.” “SOS should actively promote training and access for all.”

## Discussion

### Principal findings

This national survey provides the first quantification of point-of-care ultrasound (POCUS) use among GPs working within SOS Médecins, a nationwide network delivering urgent and continuity of primary care in France. Nearly half of the respondents (47.9%) reported using POCUS in their current practice. POCUS adoption was lowest among the youngest and oldest GPs, and highest in intermediate age groups—particularly among those aged 36 to 55. Among users, the tool was primarily employed for the evaluation of acute abdominal pain, vascular assessments and respiratory symptoms. Most used portable devices and performed targeted exams based on specific clinical suspicions.

Non-users primarily cited lack of specific training, time constraints, and equipment cost as key barriers. Notably, more than half of the non-users expressed interest in POCUS training.

These findings highlight an advanced but largely informal diffusion of POCUS in urgent general practice, alongside persistent structural constraints limiting its broader and safer integration.

In both user and non-user groups, the lack of financial recognition emerged as a recurring concern, a persistent gap between clinical practice and the current regulatory and billing framework in France.

### Comparison with existing literature

When interpreted in light of international data, and within the context of a self-selected sample, the adoption level observed in our study appears relatively high for a primary care setting. In Denmark, a recent national survey found that 11.5% of GPs reported active POCUS use [21]. While the methodological contexts differ, particularly regarding response rates, this comparison highlights the notable diffusion of POCUS within the SOS Médecins network. In Hong Kong, a cross-sectional evaluation of primary care physicians reported a prevalence of 22.5% [22].

In Switzerland, a nationwide study combining billing data and survey responses found that 49% of GPs had billed at least one ultrasound examination between 2004 and 2018. While this figure does not reflect regular or systematic use, the authors reported a steady increase in the number of users and procedures over time, suggesting a progressive diffusion of ultrasound into primary care settings [16]. More recently, Zumstein et al. (2024) found that 56.5% of Swiss GPs owned an ultrasound device, and 77% of reported scans were self-performed, particularly in rural or urgent care settings, underscoring the relevance of POCUS in areas with limited access to diagnostic imaging [6].

Although few international studies provide precise or methodologically comparable prevalence, several investigations support the growing integration of POCUS in primary care. Kornelsen et al. reported frequent use and positive experience with POCUS among rural family physicians in British Columbia, Canada, underlining its relevance in remote or resource‑limited settings [23]. By contrast, uptake appeared more limited in rural settings within the French context, with a minority of users practicing in rural areas, reflecting potential geographic inequalities in access to POCUS. This result should be interpreted in light of the fact that SOS Médecins operates predominantly in urban and peri-urban areas, which partly explains the low percentage of rural practitioners in our sample. This disparity nonetheless underscores the need for targeted implementation strategies in underserved or resource-limited regions.

In Norway, Myklestul et al. analysed national health‑insurance reimbursement data and found that the number of GPs performing POCUS increased from 479 in 2009 to 2 078 in 2016; the number of registered scans rose from 8 962 to 55 921 over the same period. By 2016, about 30% of Norwegian GPs had billed for POCUS, although three out of four scanning GPs performed fewer than 10 scans per year [7].

Our finding of a U-shaped distribution in POCUS use—with lower adoption among the youngest and oldest GPs, and higher uptake among those aged 36 to 55—may reflect generational differences in exposure and training. In the United States, POCUS use varies across generations, with lower uptake among senior GPs trained before its integration into primary care education, supporting a generational effect rather than age-related reluctance [24]. Conversely, European data suggest that younger GPs are highly motivated to integrate ultrasound into practice, even when early familiarity remains limited. For example, a Hungarian study reported strong interest among newly graduated GPs, despite nearly 60% acknowledging insufficient knowledge of indications and technique [25]. Taken together, these findings support the hypothesis that POCUS adoption is primarily driven by cumulative exposure and training opportunities across the career trajectory, rather than by age itself.

Taken together, these findings suggest that training plays a central role in POCUS adoption. Consistently, lack of training, time constraints, and equipment cost were the main barriers identified, also reported in international investigations. In a U.S. primary care–oriented survey, Nathanson et al. (2023) identified insufficient training, equipment cost, and limited funding as the main obstacles to POCUS adoption [26]. Similar barriers were reported in Australia, where high device cost and lack of reimbursement hindered implementation [27], and in Japan, where limited training opportunities remained a major constraint [28]. Even in hospital-based settings, Resop et al. (2025) observed consistent constraints across specialties, particularly regarding training and infrastructure support [29].

Beyond the identification of barriers, several studies have examined how training pathways may facilitate POCUS adoption and sustained use in primary care. Ben Shitrit et al. (2025) showed that a focused abdominal POCUS training program significantly improved primary care physicians’ skills, confidence, and frequency of use [30], supporting the central role of targeted education.

In the absence of formal curricula, Andersen et al. (2022) highlighted how Danish GPs rely on self-directed learning and peer-to-peer mentorship to acquire ultrasound competence [31].

In France, emergency physicians have developed structured POCUS training pathways and defined skill standards [32], facilitating safer and more homogeneous integration. By contrast, general practice still lacks mandatory ultrasound training, resulting in widespread informal use. This pattern aligns with the concept of “hidden integration” of POCUS in European primary care described by Sorensen et al. [33].

Beyond perceived impact, available literature suggests that POCUS use in primary care may indirectly influence patient outcomes by improving diagnostic accuracy, reducing diagnostic uncertainty, and facilitating more appropriate patient orientation, particularly in acute presentations [4, 5]. Several observational and pre–post studies have reported changes in clinical management and reduced reliance on additional imaging or referrals following POCUS implementation [30, 33].

### Strengths and limitations

This is the first national-level study to investigate both the prevalence and practical modalities of POCUS use among GPs working in French urgent primary care. Its main strengths include the inclusion of both users and non-users, the analysis of perceived barriers, impact, and training needs, as well as the study’s national scope. The population was clearly defined, focusing exclusively on GPs working within the SOS Médecins network, which ensures homogeneity in practice context and enhances the interpretability of findings within urgent primary care.

Limitations include the response rate (16%), which may raise concerns regarding potential selection bias. In particular, clinicians who are indifferent to or opposed to POCUS may have been less likely to participate, potentially inflating the proportion of reported users. Although a selection bias cannot be excluded, the balanced distribution between users and non-users supports the internal consistency of the findings. Additionally, key demographic variables (age, gender, experience) were broadly distributed. To maximize participation and reduce sampling bias, the survey was disseminated through multiple channels, including email communications and promotion at a national congress. While the response rate remains modest, the internal balance supports the credibility of the findings. Still, we acknowledge that this participation rate limits the generalizability of our findings to all SOS Médecins GPs or to GPs in family medicine. Accordingly, comparisons with international prevalence data should be interpreted with caution, as differences may partly reflect response patterns rather than population-level variation. Because some survey questions allowed multiple answers, certain reported frequencies may overlap, and total percentages may exceed 100%. The study also highlights a regulatory grey area, as several respondents reported using POCUS routinely without access to formal training pathways, institutional validation mechanisms, or dedicated billing frameworks. In France, there is currently no requirement for GPs to hold a specific certification to conduct ultrasound examinations, as long as they consider themselves competent and can justify it in case of litigation, in line with current medico-legal standards. This situation is comparable to other procedures commonly used in primary care, such as ECG, which are often self-taught and performed without formal accreditation.

### Implications and future directions

POCUS appears increasingly embedded in the diagnostic routines of urgent primary care GPs in France. The strong adoption rate of POCUS, combined with the high level of interest in further training among both users and non-users, underscores its perceived value and relevance.

To support safe and sustainable integration of POCUS in primary care, future initiatives should focus on the following complementary levels of action:

1. Developing structured undergraduate training and defining core competencies for general practitioners. Integrating ultrasound education into early medical training is essential to prepare future GPs for the evolving scope of clinical practice. National curricula should clearly define the competencies relevant to general practice and be informed by robust evidence on the utility and safety of POCUS in unselected primary care populations [33].;
2. Expanding continuing medical education opportunities for practicing GPs. Short-format, high-yield training programs focused on common and high-impact indications (e.g. abdominal pain, dyspnea, trauma) have shown strong potential to improve diagnostic confidence and clinical decision-making in primary care contexts [9]. These should be made more widely accessible and standardized.
3. Fostering local initiatives within the SOS Médecins network. Internal training, structured mentorship, and collaborative image review activities—whether formal or peer-led—can support the development and maintenance of skills. Such initiatives could be implemented both locally (within each association) and nationally to build a shared culture of quality and safety.
4. Enhancing financial and administrative recognition. Administrative barriers remain significant. Improving awareness of existing billing codes and advocating for clearer, dedicated reimbursement mechanisms are essential to ensure equitable access and long-term integration [34].

Further studies are needed to assess the clinical and cost-effectiveness of POCUS in French primary care, including its effects on referral rates, diagnostic delay, and patient outcomes. Implementation research should also explore the feasibility of regional mentorship networks or hospital-GP collaborations to foster real-time learning and quality control, as suggested by prior studies linking POCUS to reduced diagnostic uncertainty and healthcare utilization [35].

Although this study focused on GPs working in urgent and unscheduled care, its findings raise broader questions about the potential use of POCUS in family medicine. Several facilitating factors identified here, such as perceived diagnostic value, clinical autonomy, and professional satisfaction, are likely shared by GPs in family medicine. In the context of growing medical deserts, where access to specialist consultations or imaging facilities is often limited, POCUS could play a valuable role in enhancing diagnostic equity and reducing avoidable delays.

## Conclusion

This national survey provides the first detailed insight into the use of POCUS among GPs working in urgent primary care within the SOS Médecins network. With an adoption rate of 47.9% among respondents, our findings highlight a substantial but heterogeneous uptake, particularly influenced by GPs age, training background, and access to equipment.

While the overall response rate (16%) limits generalizability, the balanced representation of both users and non-users suggests that these results offer a credible reflection of current trends and perceptions within this unique out-of-hospital care model. The strong training demand among non-users and the positive perceptions reported by users indicate a clear momentum toward broader and safer integration of POCUS in French primary care.

Policymakers and professional bodies should now focus on developing and structuring undergraduate POCUS education within medical schools, expanding accessible continuing training programs, improving awareness and usability of existing billing pathways, establishing appropriate reimbursement frameworks, and fostering local mentorship initiatives to ensure safe and sustainable implementation.

Further research should evaluate the clinical and economic impact of POCUS in primary care.

## Data Availability

Deidentified data may be available in French from the corresponding author on reasonable request.

## Abbreviations

GDPR: General Data Protection Regulation
GP: General practitioners
POCUS: Point-of-care ultrasound
